# On the Treatment of Dysentery

**Published:** 1851-07

**Authors:** John Hardin

**Affiliations:** Louisville, Ky.


### WESTERN JOURNAL

OF

### MEDICINE AND SURGERY.

JULY, 1851.

ART I.—*On the treatment of Dysentery.* By JOHN HARDIN, M. D., of Louisville, Ky., A letter addressed to the editors of the West. Jour. Med. and Surg.

As the season for Dysentery is approaching and as we have already "an earnest" of it in our city, permit me in a rambling epistolary style, to give the results of two different methods of treatment which have proven highly satisfactory within the field of my observations. I do this the more cheerfully and with less diffidence, because I claim no originality either in the remedies or the manner of using them. The two methods may be denominated the Saline and the Terebinthinate. The former I derived from Dr. T. Q. Walker, of Green Co., Ky.—the latter from Dr. A. M. Allen, of Shelbyville, both of them gentlemen whose high professional and scientific attainments are freely acknowledged by all who have enjoyed the pleasure of their acquaintance.

As the diagnosis, pathology and morbid anatomy of flux are so universally understood, I will not encumber this communication with any remarks in relation to them. As the remote cause or causes are not well understood, I will not trouble you with unprofitable speculations in regard to them. As atmospheric vicissitudes and crude, indigestible ingesta are universally known to be potent exciting causes, every body is aware that the former should be guarded against as much as possible, and the latter wholly avoided; therefore I need not administer any admonitory cautions on those points. These matters being so summarily disposed of leaves me nothing farther to do but to pass at once to the subject of this letter, viz: the treatment.

The most widely diffused method of treating dysentery has been, and still is by mercurials, opiates and astringents, in multifarious forms and combinations. In addition to these, authors recommend bloodletting, but so far as I have observed, this is not often practised without an unequivocal demand for it by the state of the pulse, and this, we do not often find in this disease.

*Mercurials.*—The first objection that I make to this treatment is, that in flux, mercurials irritate an already inflamed surface, whether they excite biliary secretions or not. If they act as purgatives *per se*, they are irritant purgatives; if they procure a discharge of black bile, some idea may be formed of its effects in passing over an inflamed surface, by recalling to mind its frequent excoriations of the skin around the anus, where it has the protection of the scarf skin—how much more intense its action on an inflamed and abraded surface! Every observant practitioner knows that *good bilious* discharges are no guaranty against the recurrence of those that are *muco-sanguinolent.* An observation far from limited, has satisfied me that the action of this class of remedies in flux is pernicious. Reflection has suggested the above explanation as the reason of this deleterious action. I therefore conclude that the mercurial plan recommended by authors has been too long and too implicitly followed.

*Venesection.*—I have sometimes heard physicians say they could not bleed, because the pulse is too feeble. Marshall Hall directs that in cases of doubt respecting the propriety of bloodletting, the patient should be placed erect or sitting and bled in that posture. I have derived a different and I think a useful lesson in the employment of this remedy in dysentery. This rule is in recent cases to bleed the patient in the recumbent posture, so as to detract as much blood as can be borne, with the intention of subduing a dangerous inflammation as speedily as possible. The inquiry should be, *will the patient tolerate the loss of blood?* not, does the apparent state of the circulation demand it? If it can be tolerated, then surely it is imperatively demanded in an acute inflammation so extensive, so dangerous, and hastening so rapidly into disorganization. Without it all other remedies will too often fail.

*Treatment by Salines.*—This consists in the administration of two or three scruples of epsom salts, (a level tea spoonful) after each muco-sanguinolent discharge, no matter how frequently repeated, until clear serous evacuations are obtained devoid of mucus or blood, thus depleting from the engorged capillaries at the seat of inflammation. As soon as such evacuations are obtained, laudanum or morphine may be administered either by the mouth or rectum, after which it is not unusual for a patient to remain quiet without evacuation for six, eight or ten hours, or until such time as the inflamed capillaries become re-engorged. During the summer and autumn of 1849, I witnessed the treatment of more than one hundred cases upon this plan, with results the most gratifying, there occurring only five deaths, and but one of these had the benefit of this treatment from the beginning of the disease.

In the summer of 1850 I had the opportunity of con trasting this with yet another plan, which brings me to a consideration of the

*Terebinthinates.*—To this plan, after a most careful series of observations, I award the preference. This, like the saline treatment, commences with V. S. in all cases that will tolerate it, followed by spts. turpentine in doses of a tea spoonful every three or four hours to an adult. If it is desired to evacuate the bowels, one or two teaspoonfuls of castor oil should be added. Should there be no acrid, offensive matter to be carried off, the turpentine is given alone for the purpose of coming in contact with the inflamed surface, either by passing directly over them or reaching them through the medium of the circulation. For the tenesmus and tormina, morphine either by the mouth or rectum should be used according to circumstances. This method I do not hesitate to affirm, is the most speedy and safe that I have seen used. In both the seasons, (1849 and 50,) in which these observations were made, the disease was in a malignant epidemic form. In the last named year it prevailed concurrently with cholera. The only inconvenience that I have seen attend the use of turpentine, was the occassional, though not frequent occurrence of dysury, hematuria or strangury; these, however, soon subside, requiring little else than a suspension of the remedy. Of course under all modes of treating flux, abdominal fomentations, poultices, &c., are serviceable, and if you will not suspect me for having been raised in the *pine woods*, I would suggest, that a poultice terebinthinated over its whole surface, is the very best of that class of remedies.

Although salines and terebinthinates are not new remedies in flux, yet the manner of their use herein indicated as *the leading curative agents*, will, I think, be found a new and useful application of old remedies.

In the Medical Examiner for 1850 we find an excellent article by Dr. S. Jackson, on the Oil of Turpentine in

Typhus Fever. In that article occurs, as it were incidentally, the following passage:

"In the cachectic state of dysentery the very same advantages are obtained from turpentine, but here a very different state of things obtains; in idiopathic fevers, there is something to contend with besides the inflammation, and therefore, this stimulus cannot be used till the phlogistic diathesis be much on the wane; but in dysentery the local disease is paramount, and since this is concentrated in a mucous membrane and has no tendency to a translation, and more particularly as the oil seems peculiarly suited to inflammation of this very organ, it may be given rather early in the disease. Therefore, though its energy appears most conspicuous in the cachectic state, it will be found eminently successful in preventing this state, if given early with all the necessary co-adjuvants.

"Nor is it to be neglected as an external remedy. When diligently rubbed on the abdomen, it is soon perceived in the breath and urine; and of course it is carried to the diseased membrane. I have thus profited much by it in cases where leeches and blisters were not thought requisite." The whole of this article is highly interesting and instructive, and would render good service to the profession if a copy of it was in the hands of each of its members. Indulge me with another extract from Braithwaite's Retrospect, part xxi, on the treatment of diarrhea, by oil of turpentine, by Dr. J. J. Trayer.

"Dr. Trayer speaks highly of this medicine which he used extensively in the diarrhea which prevailed in his locality at the time when cholera was so prevalent in other parts of the island. The disease was marked by a very sudden invasion after exposure to cold, or the indigestion of some noxious article of diet; the stools were fluid, often tinged with blood, very frequent and exhausting; there was very little abdominal pain, but distressing pain and sense of fatigue in the loins and lower extremities; and there was frequent vomiting. With respect to the treatment, Dr. Trayer says: the medicine which I use as a sheet anchor in these cases is the rectified spirits of turpentine, in small doses (from 5 to 20 minims) repeated more or less frequently, and variously combined as the circumstances of the case demand; the most usual combinations being the tincture of opium.

"In a case of average intensity my mode of proceeding is this:—If my patient is still up, I order him at once to bed, and apply a light, but large bran poultice, moistened with infusion of chamomile, and occasionally sprinkled with laudanum, over the abdomen. This being covered with oiled silk, maintains its warmth and moisture for a long time. Warm jars placed to the feet, a liberal supply of bed clothes, and a warm but not close atmosphere in the bed room, render great assistance. A dose of spirits terebinth. about mxv, is given combined with laudanum, or if this be for any reason contra-indicated, an equivalent of some other sedative (Hyosciamus e. g.); or sometimes without any other medicament. This dose is felt almost immediately to check the disease; and here, if I were to describe my own feelings, the aptest expression I could use is, that a message seems as by electric telegraph to be conveyed on the moment to the whole canal, that the discharges are to cease. In place of the sickly nausea, of the coldness and sensation of incessant movements of the bowels, which tell you that your serum is oozing out, a feeling of warmth and cheerful tone at once pervades the system, and tells you that the disease is checked: in fact the first or second dose would generally complete the cure if the patient would remain in bed a few hours, and for a day or two observe a strict dietary. If the process be not always so speedy, still perseverance in the use of these measures, has, as yet, succeeded in every case in which I have tried them with one exception."

The most convenient vehicle that I have seen used for the administration of turpentine is sweet milk.—a tea spoonful of the former to a table spoonful of the latter.

June 12, 1851.

